# Unoperated severe aortic stenosis: decision making in an adult UK-based population

**DOI:** 10.1308/003588412X13171221591817

**Published:** 2012-09

**Authors:** AA Badran, HA Vohra, SA Livesey

**Affiliations:** Wessex Cardiac Centre, Southampton General Hospital,UK

**Keywords:** Aortic valve stenosis, Aortic valve, Heart valve disease, Echocardiography, Heart valve prosthesis implantation, Cardiovascular surgical procedures

## Abstract

**INTRODUCTION:**

Severe symptomatic aortic stenosis is associated with a poor prognosis, with most patients dying 2–3 years after diagnosis. We analysed the proportion of patients with severe aortic stenosis not referred for aortic valve replacement (AVR) in a UK-based population and the clinical factors contributing to this.

**METHODS:**

Retrospective analysis of patients with echocardiographic evidence of severe aortic stenosis was performed at a university teaching hospital.

**RESULTS:**

A total of 178 consecutive patients with severe aortic stenosis (AVA: <1cm^2^, mean pressure gradient: ≥40mmHg, or visually severe on echocardiography) were included in the study. Eighty-three patients did not have AVR (95% confidence interval: 39–54%). The cohort included 146 symptomatic patients (82%) and 32 (18%) who were asymptomatic. The most common reason for non-referral in symptomatic patients was ‘high operative risk’ and in asymptomatic patients ‘no symptoms’. Of the patients who did not have AVR, only 19% (*n*=16) were referred for a surgical opinion. None of the patients in the asymptomatic group underwent echocardiographic stress imaging. The thirty-day operative mortality rate in the AVR group was 2.3%. Symptomatic patients who underwent AVR had superior survival, even after adjusting for co-morbidities (*p*<0.001).

**CONCLUSIONS:**

A considerable proportion of patients with severe aortic stenosis are not referred for surgery although they have a clear indication for AVR. Patients are often estimated as being too high risk or having prohibitive co-morbidities. Among asymptomatic patients, stress imaging was rarely used despite its useful role prognostically and in deciding the best time for intervention.

In the developed world aortic stenosis (AS) is the most common valve disease requiring surgery.^1^ It is predominantly a disease of older age, with a prevalence of 5.2% in people >75 years.^2^ Due to a continuously ageing western population, AS constitutes a growing health burden.^2^ The natural history of AS is well established, characterised by a long asymptomatic period that is variable between individuals.^1,3^ Onset of symptoms occurs late in the disease and is associated with an ominous prognosis, with a high incidence of sudden death.^1,4^

Echocardiographic stress imaging has been recommended as an effective but underused way of investigating the symptomatic status of disease in severe AS.^3,5^ Symptom precipitation on stress imaging is itself an indication for surgery according to guideline recommendations.^3,6^ Guidelines also advocate intervention in some but not all asymptomatic patients, with consideration for stress imaging to help stratify risk.^3^ In some asymptomatic patients, avoiding irreversible myocardial damage in the wait for symptoms is the rationale for surgical intervention.^7^

## Methods

Patients with a diagnosis of severe AS were identified retrospectively from the echocardiography database of our university teaching hospital. Severe AS was defined according to European Cardiac Society guidelines (mean pressure gradient [MPG] ≥40mmHg or aortic valve area <1cm^2^) in addition to patients identified as being severe visually on echocardiography. The latter criterion was included because parameters such as MPG are largely dependent on normal systolic function and the existence of ventricular dysfunction would therefore lead to a gradient that is underestimated relative to a heart with normal systolic function.

Between July 2008 and March 2010, 184 consecutive patients underwent echocardiography interpreted to be consistent with severe AS according to the inclusion criteria. Five patients were excluded because they had a previous aortic valve replacement (AVR). This was to maintain generalisability of the results. One patient was found not have severe AS after stress imaging.

The medical records were used to gather demographic information, echocardiographic parameters, referral status, details of surgery performed, calculated logistic EuroSCORE (http://www.euroscore.org/), reasons for non-referral, symptomatic status, co-morbidities and follow-up information.

## Results

The characteristics of the 178 patients are shown in Table 1.

**Table 1:** Characteristics of 178 patients with severe aortic stenosis

Factors	Decision to operate (*n*=95)	Decision not to operate (*n*=83)	*p*-value
Mean age (range)	77.19 (51–97)	81.89 (49–96)	**<0.001**
Age ≥80 years	41 (43%)	57 (69%)	**0.01**
Female	49	44	0.849
Male	46	39	
Mean time to last follow up (days)	189.80 (SD: 182.42)	186.84 (SD: 195.45)	0.294
Symptoms	87	59	**0.002**
No symptoms	6	18	
Unknown symptom status	2	6	
No/unknown symptoms and stress tested	0	1	
Median logistic EuroSCORE	5.09 (IQR: 3.94–8.37)	7.40 (IQR: 4.60–11.79)	**0.002**
Active cancer	0	4	**0.030**
Chronic kidney disease	4	11	**0.030**
Congestive heart failure	1	8	**0.009**
Prior cardiac surgery	7	5	0.721
Chronic lung disease	2	10	**0.008**
Diabetes	17	9	0.184
*Echocardiographic factors*			
Mean MPG (mmHg)	46.80 (SD: 20.23)	39.12 (SD: 16.39)	**0.015**
Mean PPG (mmHg)	78.63 (SD: 32.14)	65.10 (SD: 24.76)	**0.004**
Mean aortic valve area (cm^2^)	0.61 (SD: 0.19)	0.63 (SD: 0.22)	0.703
Mean LVEF (%)	58.73 (SD: 21.04)	56.93 (SD: 16.95)	0.662
Mean LVID – diastole (mm)	4.75 (SD: 0.83)	4.69 (SD: 0.91)	0.935
Mean LVID – systole (mm)	3.05 (SD: 0.98)	3.20 (SD: 0.91	0.470
Mean LVPW thickness (mm)	1.34 (SD: 0.31)	1.26 (SD: 0.26)	0.262
Mean IVS thickness (mm)	1.49 (SD: 0.436)	1.36 (SD: 0.34)	0.172

SD = standard deviation; IQR = interquartile range; MPG = mean pressure gradient; PPG = peak pressure gradient; LVEF = left ventricular ejection fraction; LVIDD = left ventricular internal dimension; LVPW = left ventricular posterior wall; IVS = interventricular septum

### Referral and stress imaging

Ninety-five patients (53.4%) underwent AVR. Of these, 89 had open procedures and 7 had transcatheter aortic valve implantation [TAVI], of which 1 failed and was converted to an open procedure. Eighty-three patients (46.6%) were unoperated (95% confidence interval [CI]: 39.4–54.0%). In total, 146 patients (82%) were symptomatic, 14 (7.9%) were not and in 8 (4.5%) there was insufficient information to determine symptomatic status. Seven patients were scheduled for intervention at the time of data collection. Only 16 unoperated patients (19%) were referred for a surgical opinion (95% CI: 10.2–24.7%).

Patients in the AVR and unoperated groups had similar sex profiles. Unoperated patients were older, had a higher perioperative mortality risk (logistic EuroSCORE), more co-morbidities and were more likely to have symptomatic disease. Comparison of the echocardiographic details (Table 1) between the two groups revealed a significantly lower MPG in the unoperated patients and a lower mean left ventricular ejection fraction that was not statistically significant. When excluding patients with significant co-morbidities in both groups, echocardiographic parameters were similar. The unoperated group, however, was older (*p*<0.001), had a higher logistic EuroSCORE (*p*=0.026) and more asymptomatic disease (*p*=0.015).

Of the 83 unoperated patients, 63 (76%) were evaluated by a cardiologist and 16 (19%) by a cardiac surgeon. Of the asymptomatic patients or those who had asymptomatic status as the reason for not having AVR (*n*=10), none underwent stress imaging.

### Analysis of decision to operate

Univariate analysis showed that patient age, significant co-morbidities, logistic EuroSCORE, MPG and symptomatic status all significantly affected the decision to operate (Table 1). In multivariable analysis (Table 2), however, the logistic EuroSCORE did not reach statistical significance (*p*=0.819).

**Table 2:** Factors associated with a decision not to operate

Factors	*p*-value	Odds ratio	95% confidence interval
Age	**0.02**	0.910	0.841–0.984
<75 years	0.888	1.097	0.301–3.999
75–85 years	**0.012**	3.515	1.322–9.342
>85 years	**0.024**	0.261	0.810–0.840
Mean pressure gradient	**0.002**	1.055	1.020–1.091
Logistic EuroSCORE	0.819	0.984	0.860–1.127
Symptomatic status	**0.03**	4.530	1.200–17.077
Significant co-morbidities	**0.007**	0.149	0.037–0.596

### Symptoms associated with aortic stenosis

Most patients (*n*=146) had symptomatic severe AS. Of the 83 unoperated patients, 59 were symptomatic and 24 were asymptomatic or had unknown symptomatic status. Patients with a history of chronic lung disease and only dyspnoea as a symptom were not regarded as being symptomatic. Among unoperated patients, symptomatic patients were older (mean: 83.3 years, standard deviation [SD]: 8.83 years) than asymptomatic patients (mean: 77.4 years, SD: 8.29 years) (*p*=0.008).

### Operative risk

The predicted perioperative mortality risk was lower for operated than for unoperated patients (*p*=0.002) and also lower among unoperated asymptomatic patients (median: 4.26%) compared to unoperated symptomatic patients (median: 7.87%) (*p*<0.001).

The median logistic EuroSCORE in the group of 59 symptomatic patients not undergoing AVR was 7.87% (interquartile range [IQR]: 6.20–12.06%). More than two-thirds (*n*=38, 70%) had an estimated perioperative mortality risk of ≤10%. Among symptomatic patients with severe AS who underwent AVR, 73 (85%) had an estimated perioperative mortality risk of ≤10%.

Of the 83 unoperated patients, 25 (30%) had a calculated operative risk of no more than the median calculated risk among patients who underwent AVR (5.09%).

### Rationale for conservative management

**Figure 1:**
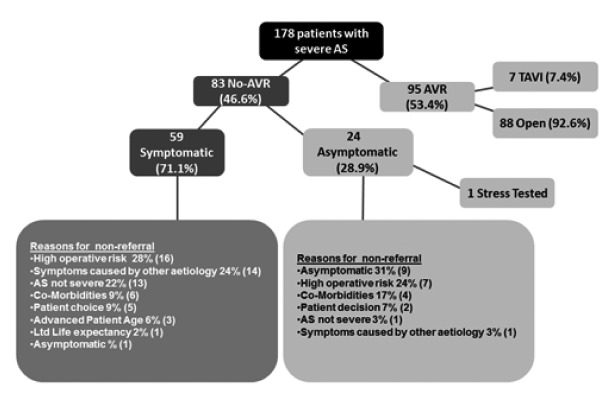
Flow diagram of the 178 patients with severe aortic stenosis

For patients who did not undergo AVR, decisions not to refer for surgery were based on several factors (Fig 1).

Calculated perioperative risk was the highest among patients who were unoperated due to ‘advanced age’ and a ‘perceived high operative risk’ (median logistic EuroSCORE of 11.99% and 10.35% respectively).

Among the 14 unoperated symptomatic patients in whom symptoms were attributed to an aetiology other than AS, exercise stress imaging to evaluate the impact of AS on cardiac function was performed in 1 patient. In those with asymptomatic status as the main reason for not having AVR, none were stress tested.

### Impact of advanced age

Among all 178 patients with severe AS, 98 (55%) were over 80 years of age, including 41 patients (43%) who underwent AVR and 57 (69%) who were unoperated (*p=*0.01). Age >80 years was associated with a higher logistic EuroSCORE for both operated (median: 6.54%, IQR: 5.09–10.86%) and unoperated patients (mean: 7.87%, IQR: 6.45–12.80%). In addition, the calculated perioperative mortality risk among patients over 80 years of age was higher for unoperated than for operated patients (*p*=0.01).

### Impact of co-morbidities

Among all 178 patients with severe AS, 35 (20%) had significant co-morbidities including congestive heart failure, chronic kidney disease, chronic lung disease and cancer. After excluding patients with these co-morbidities, age (*p*<0.001), EuroSCORE (*p*=0.026) and symptomatic status (*p*=0.015) were the only significant differences between the two groups.

### Follow up

One-year follow-up data were available for 146 patients (82%), 79 of whom had a decision to operate and 67 of whom had a decision not to operate (*p*=0.086). The mean follow-up duration for patients referred for surgery was 186.84 days (SD: 195.45 days, range: 0–742 days). A total of 34 unoperated patients died including 25 symptomatic patients, 8 asymptomatic patients and 1 patient of unknown symptomatic status. In patients not referred for surgery the mean follow-up duration was 189.80 days (SD: 182.42 days, range: 0–723 days). Five patients died; all were symptomatic. One additional (symptomatic) patient died during the follow-up period while awaiting surgery.

**Figure 2:**
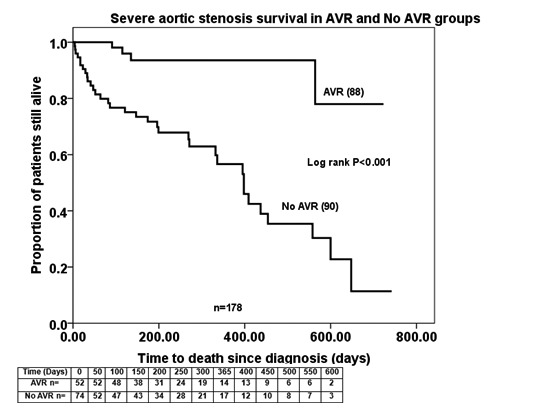
Severe aortic stenosis survival rates in aortic valve replacement (AVR) and unoperated groups

**Figure 3:**
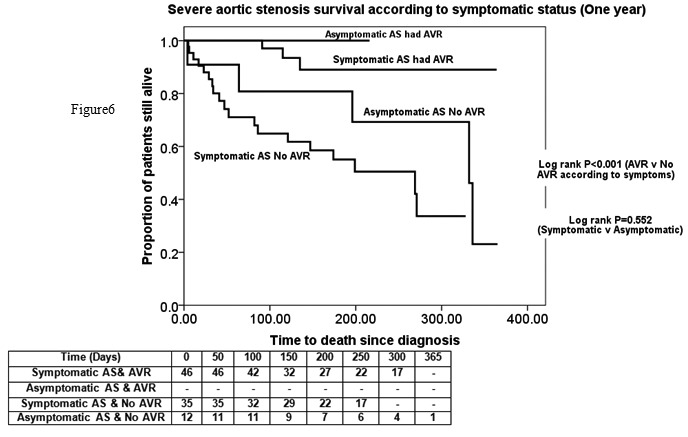
Severe aortic stenosis survival rates according to symptomatic status (one year)

Survival rates are shown in Figs 2 and 3. One- and two-year survival following diagnosis was 97.1% and 96.3% respectively for the AVR group and 76.9% and 75% respectively for the conservative group. Survival was not found to be statistically different based on symptomatic status (*p*=0.552) but symptomatic disease was associated with a lower survival in those operated and unoperated (*p*<0.001). Survival analysis with significant co-morbidities and age >85 years excluded still showed the difference in survival (*p*=0.005).

In multivariable analysis (Table 3), the decision to operate (AVR), patient age and significant co-morbidities (chronic lung disease and chronic kidney disease) were linked to the outcome. When excluding significant co-morbidities, only AVR was linked to the outcome, with those aged 75–85 years deriving the most benefit (odds ratio [OR]: 12.256, *p*=0.004) and those aged <75 years (OR: 11.232, *p*=0.004) and >85 years (OR: 10.921, *p*=0.006) deriving a significant but lesser improved survival from surgery.

**Table 3:** Factors associated with a decision not to operate**(significant co-morbidities excluded)

Factors	*p*-value	Odds ratio	95% confidence interval
Age	0.400	0.919	0.848–0.996
<75 years	0.888	1.097	0.301–3.999
75–85 years	**0.027**	3.167	1.141–8.790
>85 years	**0.024**	0.261	0.810–0.840
Mean pressure gradient	**0.012**	1.049	1.010–1.089
EuroSCORE	0.819	0.984	0.860–1.127
Symptomatic status	**0.024**	5.480	1.246–24.095

### Discussion

In this study, approximately half of the patients with severe AS did not undergo intervention, with over two-thirds being symptomatic. Therefore, 1 in 3 patients with severe AS had symptoms but were not referred for surgery.

### Impact of patient age

Age per se, however, is not a valid reason to preclude patients with severe AS from AVR,^3^ particularly when it has been well documented that surgery can be performed safely in octogenarians and nonagenarians.^9–11^ Decision making should rely not only on an estimation of operative risk but also on estimation of the risk–benefit ratio, requiring outcome after surgery to be compared with natural history. Age is a predictor of operative risk and poor late survival in cardiovascular surgery, particularly in the case of AS.^12^ Nevertheless, age is not a predictor of poor late outcome when considering relative survival.^10,12^ This led to guideline recommendations that advanced age is not a contraindication to valve replacement.^3,6^

The associated clinical implications are substantial because prognosis without intervention is dismal (in this study, one-year survival was only 77% among unoperated symptomatic patients) whereas AVR is associated with both symptomatic improvement and improved survival (in this study, 97% survival at one year).

### ‘High operative risk’?

Among symptomatic patients who did not undergo surgery, perceived high operative risk was cited most often as a reason, followed by symptoms attributed to another aetiology. While operative risk was significantly higher in unoperated patients, they were older and had more co-morbidities. Furthermore, the logistic EuroSCORE did not reach statistical significance on multivariate analysis of the decision to operate while age >75 years did.

Although some unoperated patients had very high operative risks, many symptomatic patients who did not undergo surgery appear to have had an acceptable operative risk based on objective measures: 54% had a logistic EuroSCORE <10% and in 15 patients the calculated mortality risk was less than the median risk for patients who underwent AVR (5.09%). Subjective means of assessing operative risk may be unreliable, at a cost of denying consideration for intervention to patients who are legitimate candidates and who might benefit from surgery.^13^

Reports have demonstrated that advanced age is an important factor in denying surgery for severe AS.^14,15^ In our study, age >85 years was the only significant difference preventing patients from having surgery when excluding significant co-morbidities. Although unoperated patients had a higher perioperative risk, clinically, an increase in logistic EuroSCORE of 2.31% should not deny surgery in patients with significantly poor survival if unoperated.

Less than a fifth of unoperated patients were even referred for a surgical opinion. Severe AS is a mechanical obstruction requiring surgical intervention for any hope of an effective treatment. These patients should therefore be seen by a surgeon to determine whether or not they are suitable for surgery.

### Underestimation of symptoms

The triad of severe AS symptoms (dyspnoea, syncope, angina) was documented for 33 patients but regarded as mild, non-debilitating or due to another aetiology. Having mild symptoms does not exclude a patient from being an AVR candidate.^6,8^ Furthermore, it is known that even if symptoms are recognised, the resulting functional disability is often underestimated by physicians.^8^

The poor prognosis and subtlety by which symptoms are exhibited means that stress echocardiography should be used to aid in the stratification of risk and the decision making process.^3,5^ In our series, only one patient in the non-symptomatic group underwent stress imaging after diagnosis.

### Co-morbidities

Co-morbidities are frequent in the elderly and are expected to affect the risk–benefit analysis because they influence life expectancy regardless of valvular disease as well as the operative risk and late outcome after AVR.^16,17^ While a decision to operate was less likely in patients with more co-morbidities, age remained a highly significant factor in the decision to operate when those with significant co-morbidities were excluded from the analysis.

### Patient outcome analysis

In our series, 30-day mortality was relatively low (2.3%) given the patient risk profile as reported in certain series.^15^ One-year survival was poor in unoperated patients with AS, which is in line with the poor prognosis associated with the disease and perhaps suggests that those regarded as asymptomatic in fact had underlying symptoms.

When excluding significant co-morbidities, only AVR was linked to the outcome. This reinforces that the survival benefit between the two groups can be attributed to AVR rather than age or co-morbidities.^15^

### Transcutaneous valve implantation

The advent of TAVI and its validation as a feasible treatment option in some patients deemed to be high risk make the lack of AVR in these patients even more relevant.^18^ Seven patients in the AVR group underwent TAVI as they were deemed not suitable for open surgery. Therefore, even in a centre using percutaneous AVR, many patients are still treated conservatively.

### Study limitations

This was a retrospective observational study. Nevertheless, patients with severe AS were identified in a consistent, consecutive manner compatible with guideline recommendations for AS severity.^3^ Such an observational study does not enable the appropriateness of the therapeutic decision to be fully assessed for an individual patient. However, it does enable the decision for surgery to be analysed prospectively and put into perspective with cardiac as well as non-cardiac patient characteristics in a UK-based population of patients.

## Conclusions

Data from this study suggest that many patients in a UK-based population who could benefit from surgical intervention for symptomatic severe AS do not undergo AVR. Analysis of the clinical decisions precluding AVR found that a perceived high operative risk and attributing symptoms to another aetiology are common reasons why symptomatic patients are denied surgery. A consistent finding on univariate and multivariate analysis of the decision to operate was that age plays a significant role in denying patients surgery. Most patients who do not receive AVR are not assessed by a surgeon and do not undergo echocardiographic stress imaging. A multidisciplinary approach should be taken to ensure that patients have a more informed choice of how best their disease can be managed.

## References

[1] Kurtz CE, Otto CM. Aortic stenosis: clinical aspects of diagnosis and management, with 10 illustrative case reports from a 25-year experience. Medicine (Baltimore) 2010; 89: 349–379 21057260 10.1097/MD.0b013e3181fe5648

[2] Nkomo VT, Gardin JM, Skelton TN*et al.* Burden of valvular heart diseases: a population-based study. Lancet 2006; 368: 1,005–1,011 16980116 10.1016/S0140-6736(06)69208-8

[3] Vahanian A, Baumgartner H, Bax J*et al.* Guidelines on the management of valvular heart disease: the Task Force on the Management of Valvular Heart Disease of the European Society of Cardiology. Eur Heart J 2007; 28: 230–268 17259184 10.1093/eurheartj/ehl428

[4] Otto CM. Timing of aortic valve surgery. Heart 2000; 84: 211–218 10908267 10.1136/heart.84.2.211 PMC1760905

[5] Rafique AM, Biner S, Ray I*et al.* Meta-analysis of prognostic value of stress testing in patients with asymptomatic severe aortic stenosis. Am J Cardiol 2009; 104: 972–977 19766766 10.1016/j.amjcard.2009.05.044

[6] Bonow RO, Carabello BA, Chatterjee K*et al.* 2008 Focused update incorporated into the ACC/AHA 2006 guidelines for the management of patients with valvular heart disease. Circulation 2008; 118: e523–661 18820172 10.1161/CIRCULATIONAHA.108.190748

[7] Owen A, Henein MY. Challenges in the management of severe asymptomatic aortic stenosis. Eur J Cardiothorac Surg 2011; 40: 848–850 21367614 10.1016/j.ejcts.2011.01.031

[8] van Geldorp MW, van Gameren M, Kappetein AP*et al.* Therapeutic decisions for patients with symptomatic severe aortic stenosis: room for improvement? Eur J Cardiothorac Surg 2009; 35: 953–957 19303794 10.1016/j.ejcts.2009.01.043

[9] Tsai TP, Denton TA, Chaux A*et al.* Results of coronary artery bypass grafting and/or aortic or mitral valve operation in patients > or = 90 years of age. Am J Cardiol 1994; 74: 960–962 7977134 10.1016/0002-9149(94)90599-1

[10] Krane M, Voss B, Hiebinger A*et al.* Twenty years of cardiac surgery in patients aged 80 years and older: risks and benefits. Ann Thorac Surg 2011; 91: 506–513 21256302 10.1016/j.athoracsur.2010.10.041

[11] Nwaejike N, Breen N, Bonde P, Campalani G. Long term results and functional outcomes following cardiac surgery in octogenarians. Aging Male 2009; 12: 54–57 19572233 10.1080/13685530903033224

[12] Bouma BJ, van Den Brink RB, van Der Meulen JH*et al.* To operate or not on elderly patients with aortic stenosis: the decision and its consequences. Heart 1999; 82: 143–148 10409526 10.1136/hrt.82.2.143 PMC1729124

[13] Kapadia SR, Goel SS, Svensson L*et al.* Characterization and outcome of patients with severe symptomatic aortic stenosis referred for percutaneous aortic valve replacement. J Thorac Cardiovasc Surg 2009; 137: 1,430–1,435 19464460 10.1016/j.jtcvs.2008.12.030

[14] Bouma BJ, van den Brink RB, Zwinderman K*et al.* Which elderly patients with severe aortic stenosis benefit from surgical treatment? An aid to clinical decision making. J Heart Valve Dis 2004; 13: 374–381 15222283

[15] Iung B, Cachier A, Baron G*et al.* Decision-making in elderly patients with severe aortic stenosis: why are so many denied surgery? Eur Heart J 2005; 26: 2,714–2,720 10.1093/eurheartj/ehi471 16141261

[16] Roques F, Nashef SA, Michel P*et al.* Risk factors and outcome in European cardiac surgery: analysis of the EuroSCORE multinational database of 19030 patients. Eur J Cardiothorac Surg 1999; 15: 816–822 10431864 10.1016/s1010-7940(99)00106-2

[17] Edwards FH, Peterson ED, Coombs LP*et al.* Prediction of operative mortality after valve replacement surgery. J Am Coll Cardiol 2001; 37: 885–892 11693766 10.1016/s0735-1097(00)01202-x

[18] Leon MB, Smith CR, Mack M*et al.* Transcatheter aortic-valve implantation for aortic stenosis in patients who cannot undergo surgery. N Engl J Med 2010; 363: 1,597–1,607 20961243 10.1056/NEJMoa1008232

